# Pilot study evaluating broccoli sprouts in advanced pancreatic cancer (POUDER trial) - study protocol for a randomized controlled trial

**DOI:** 10.1186/1745-6215-15-204

**Published:** 2014-06-03

**Authors:** Vladimir J Lozanovski, Philipp Houben, Ulf Hinz, Thilo Hackert, Ingrid Herr, Peter Schemmer

**Affiliations:** 1 Department of General and Transplant Surgery, University Hospital Heidelberg, Im Neuenheimer Feld 110, 69120 Heidelberg, Germany

**Keywords:** Pancreatic adenocarcinoma, broccoli sprouts, sulforaphane, palliative chemotherapy, cancer stem cells

## Abstract

**Background:**

Pancreatic ductal adenocarcinoma (PDA) is one of the most aggressive malignancies with marked resistance to chemo- and radiotherapy. PDA-cancer stem cells (CSCs) are not targeted by current therapies and may be a reason for poor prognosis. Studies indicate that diets rich in cabbage, broccoli, and cauliflower offer cancer preventative and therapeutic benefits. Recent experimental studies have confirmed these findings and demonstrated that isothiocyanate, sulforaphane, and the polyphenol, quercetin, effectively reduced tumor growth and enhanced the sensitivity of the cancer cells to current chemotherapeutics. The aim of the present study is to test the feasibility of a randomized controlled trial on the application of freeze-dried broccoli sprouts in patients with advanced PDA.

**Methods and study design:**

The study is designed as a prospective randomized, double-blinded pilot trial with a treatment and a placebo-controlled arm in a single center setting. A total number of forty patients (18 years or older) in two parallel groups with advanced, surgically non-resectable PDA under palliative chemotherapy are planned for recruitment. Patients in the treatment group will receive fifteen capsules of the study substance per day (90 mg of active sulforaphane) during the chemotherapy treatment course. Patients in the placebo group will receive the same capsule size and portion distribution with inactive substances (mainly methylcellulose). The follow-up duration is one year. Feasibility of the study substance, adverse effects, and patient compliance, as well as levels of serum tumor markers (CEA, CA 19-9), quality of life, and patient overall survival rates will be assessed at defined points of time.

**Discussion:**

The POUDER trial is expected to transfer promising experimental and epidemiological data into a clinical pilot study to assess the effectiveness of broccoli sprout extracts in the treatment of advanced PDA. The study objectives will provide data on the clinical feasibility and acceptability of a supportive treatment option accompanying palliative chemotherapy. Based on these results, future clinical studies to create further evidence in this field are possible.

**Trial registration:**

The POUDER trial has been registered at ClinicalTrials.gov with an ID NCT01879878 and WHO with an ID U1111-1144-2013 on June 13^th^ 2013.

## Background

Pancreatic ductal adenocarcinoma (PDA) is the fourth most common cause of cancer-related deaths worldwide. It is one of the most aggressive and lethal malignancies, with a 5-year survival rate of 6% in Europe and the United States, and a median survival time of 4 to 6 months [1-3]. With an incidence proportionally correlated to age [1], the numbers of estimated new cases and PDA-related deaths for 2013 in the United States alone are 45,220 and 38,460, respectively [4]. Despite standardization and advances in surgical and systemic treatment options, curative surgical intervention is possible in only 20% of the patients [5], due to the cancer’s initial asymptomatic course. Most patients present with an advanced stage of disease and can therefore only be treated in a palliative intention, which explains the nearly similar rates of incidence and mortality [1,6]. PDA-specific molecular aberrations, intense desmoplastic stroma reaction, and hypoxia are some of the factors that presumably cause the poor outcome [7].

Recent research data strongly suggest that cancer is a stem cell disease [8,9]. The small subpopulation of cancer stem cells (CSCs) within the tumor mass is thought to survive during conventional cytotoxic therapy due to its defense and survival mechanisms [10]. CSCs are believed to be capable of self-renewal and differentiation, thereby generating a heterogeneous cell population of the original tumor [8,11,12]. In addition, it has been proposed that CSCs mediate uncontrollable growth, therapy resistance, cell invasion, and metastasis [13]. Markers for CSCs have been identified in various tumor entities, including PDA, and selected marker-positive cell fractions are able to reconstitute the original tumor in immunodeficient mice [14].

In a review published in 2007, the American Institute for Cancer Research (AICR) drew attention to the benefits of increased cabbage consumption in PDA patients [15]. These results were not restricted to the pancreas, as two recent epidemiological, prospective nutrient studies demonstrated significant inhibition of metastases seeding in patients with prostate cancer [16,17]. Kirsh, *et al*. observed the significantly decreased risk of extra-prostatic manifestation of prostate cancer (stage III or IV tumors) correlated with an increase in the consumption of cruciferous vegetables, in particular broccoli which is rich in sulforaphane and quercetin (*P* = 0,02) [16]. The benefits inferred by sulforaphane, which is derived from mustard oil, and its inactive glycoside precursor, glucoraphanin (present in high concentrations in broccoli and its sprouts), have been thoroughly investigated [18,19]. Sulforaphane is believed to target CSCs through the downregulation of NF-κB [18]. This observation was first discovered in PDA and has also been confirmed for breast and prostate cancer [20,21]. Consequently, these substances may serve as an alternative treatment option for PDA; by exhausting pancreatic CSCs, the tumor sensitivity to chemotherapeutics, such as sorafenib, gemcitabine, cisplatin, doxorubicin, and 5-fluorouracil, may be significantly improved [20,22,23].

### Trial rationale

Epidemiological and animal studies in various cancer entities [15-17,20,24-29] clearly demonstrate that broccoli sprouts, rich in sulforaphane, have a tumor-suppressive potential [18]. However, to date there has been no routine clinical application of broccoli sprouts or their isolated phytochemical substances. The aim of this pilot study is to evaluate, for the first time, the feasibility and clinical impact of a daily application of encapsulated, freeze-dried broccoli sprouts in PDA patients in a palliative setting as a chemotherapy-supporting agent. The results of the study could consequently serve as the rationale for the design of further clinical trials.

## Methods/Design

The ethics committee of the University of Heidelberg has approved the POUDER trial as a monocentric, prospective, randomized, placebo-controlled clinical pilot study with two parallel groups (placebo versus verum) and provided the trial ethical approval (reference S-347/2009). Forty eligible patients receiving palliative chemotherapy, who meet the inclusion criteria (Table 1), with advanced, non-resectable PDA treated at the European Pancreas Center in Heidelberg, will be enrolled after signing informed consent. Approximately 15 to 20 PDA patients who meet the study inclusion criteria are treated in the outpatient department of the European Pancreas Center per month. Therefore, a short recruitment period of 3 to 4 months is expected. Exclusion criteria are a known intolerance to broccoli or its ingredients, impaired mental status, language problems, or a refusal of participation. Due to the nutrition supplemental character of the trial, sufficient oral intake is a prerequisite for inclusion in the study that is, the patients should not have symptoms or signs of indigestion or problems with the passage of food (nausea, vomitus, presence of nasogastric tube) (Table 1).

**Table 1:** Trial inclusion and exclusion criteria

**Inclusion criteria**	**Exclusion criteria**
Patients ≥18 years	Intolerance to broccoli or its ingredients
Advanced, surgically non-treatable PDA^a^	Impaired mental status or language problems/barriers
Written informed consent	
Intact gastric emptying	
Palliative chemotherapy	

^a^Patients who receive surgical exploration, intraoperative biopsy, and/or palliative bypass interventions (bileodigestive anastomosis and/or gastroenterostomy because of preoperative cholestasis or impaired gastric emptying due to a tumor-mass effect) will be included in this trial. PDA, pancreatic ductal adenocarcinoma.

### Trial objectives

The objective of this pilot trial is to evaluate the feasibility of nutritional supplements rich in sulforaphane and quercetin, administered via encapsulated, freeze-dried broccoli sprouts, in patients with advanced pancreatic cancer undergoing treatment with palliative chemotherapy. In this context, given that patients are supposed to take 15 capsules per day, the principal objective of this trial is to test if the daily intake is possible. In order to achieve the effective sulforaphane dose as extrapolated from our previous animal experiments, the capsules should be taken all 15 at once, however with an acceptable pause of few minutes in between.

### Outcome measures

**Figure 1:**
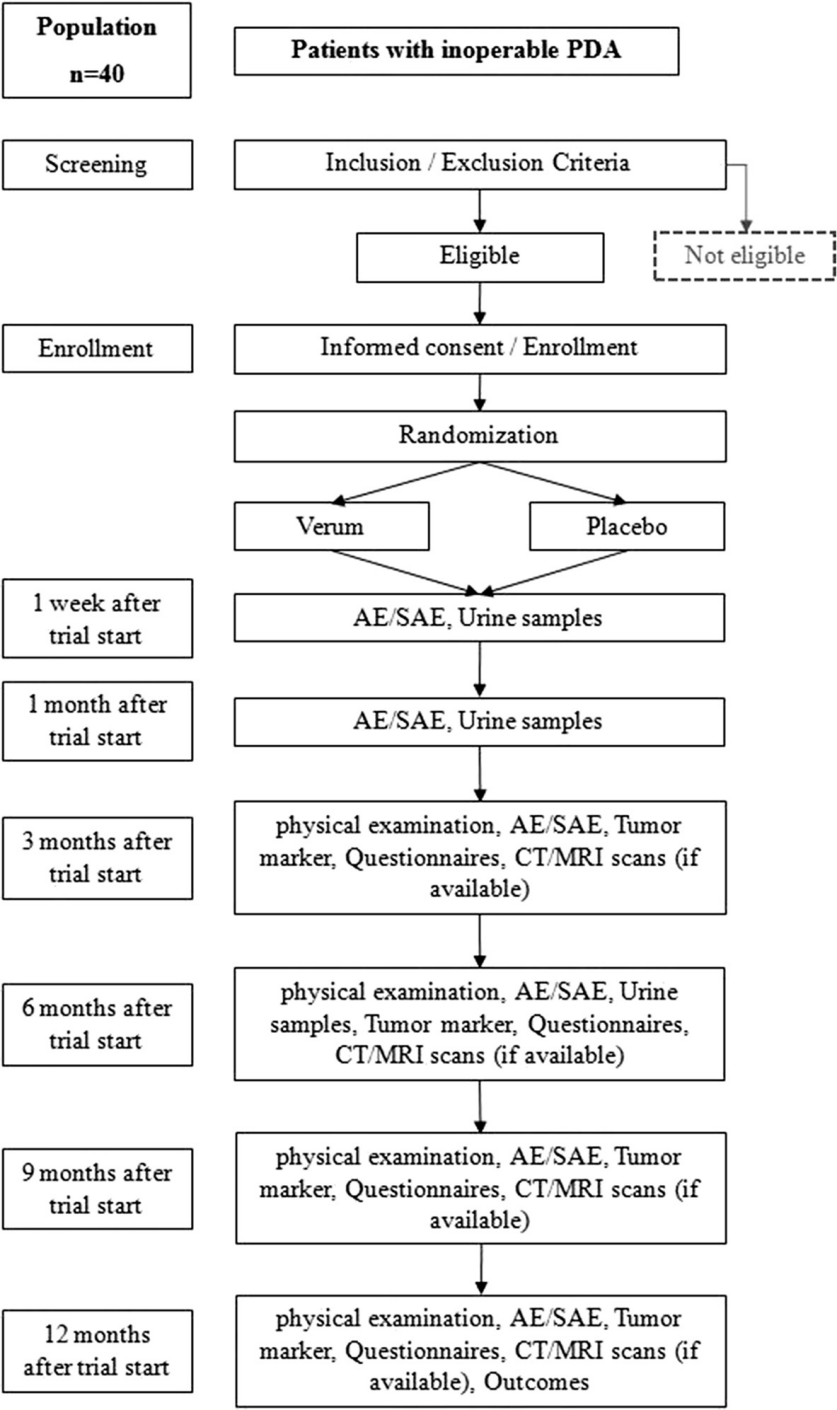
**Flow chart of the POUDER-Trial.** PDA, pancreatic ductal adenocarcinoma: AE, adverse event: SAE, serious adverse event: CT, computed tomography; MRI, magnetic resonance imaging. Tumor markers: CEA and CA 19-9.

Serum levels of the pancreatic tumor markers carbohydrate antigen 19-9 (CA19-9) and carcinoembryonic antigen (CEA), and routinely performed cross-sectional imaging (computed tomography or magnetic resonance imaging), will be monitored and assessed as parameters for evaluating disease status. Staging examinations will take place every three months after study inclusion during the treatment course with cytoreductive chemotherapy (Figure 1). The total concentration of isothiocyanates (sulforaphane) in urine will serve as a monitoring parameter for the evaluation of patient compliance. Additionally, the overall survival rate will be documented.To evaluate the quality of life of the study participants, questionnaires (European Organisation for Research and Treatment of Cancer (EORTC) Quality of Life Questionnaires (QLQ-PAN26, QLQ-C30) will be completed at every follow-up visit (3, 6, and 12 months after patient inclusion), in addition to the evaluation of a patient diary kept to document the regular intake and amount of the test substances, and any observable side effects (adverse or otherwise) (Figure 1). If outcome differences between the verum and the placebo groups are detected after completion of the POUDER trial, statistical analyses will be performed in order to generate a hypothesis for a confirmatory trial on this topic.

### Trial interventions

Patients will be given 15 capsules of either verum or placebo per day during the chemotherapy treatment course. The follow-up duration is one year. The daily dose of the nutritional supplement for the verum group contains 90 mg of active sulforaphane distributed over 15 capsules, each of which contains 400 mg of broccoli sprout grain (6 mg of sulforaphane per capsule). In contrast, the placebo capsules consist of 400 mg of inactive substances (mainly methylcellulose) with an identical capsule size and intake protocol. Study substances (for the verum and placebo groups) will be produced, labeled, and packed by Food-Service, Deiters & Florin GmbH (Hamburg, Germany). The guidelines for Good Manufacturing Practice (GMP) have been adhered to at all times. Participants will be provided with a 3-month supply of capsules at every visit (Figure 1).

Urine samples will be collected one week and one and six months after randomization to analyze the concentration of the isothiocyanates (Figure 1). The analysis will be done from frozen aliquots using 1,2-benzenedithiol cyclocondensation as described by Shapiro *et al*. [30] and assessed by high performance liquid chromatography (HPLC). The urine concentration of the degradation products of sulforaphane is used to evaluate the bioavailability of the substance after oral ingestion, as well as patient compliance. In general, sulforaphane levels are significantly higher than those of its inactive precursor, glucoraphanin, thanks in part to the nature of the precursor itself. Both sulforaphane and glucoraphanin are present in known concentrations in the administered broccoli sprouts. However, due to its slower clearance from the body [31], glucoraphanin acts as a depot for the active substance. This, in turn, prolongs the half-life of sulforaphane in the blood. By using HPLC, the conversion of the precursor to the active substance in the body can be measured and monitored.

Biochemical analysis of serum tumor markers will take place during the first visit and for all subsequent visits, scheduled at 3-month intervals, until the end of the follow-up period. Diagnostic imaging (CT/MRI scans) will be routinely performed during the oncological follow up of the patients. If the findings of the imaging diagnostic are available, they will be included as monitoring parameters and, together with the tumor markers, will provide insight into the status of the disease. The last visit will be scheduled one year after the beginning of the trial and will serve to investigate the clinical status of the patient, as well as to compile all efficacy and safety data.

### Randomization and blinding

A 1:1 randomization will be performed and patients will be assigned with a randomization number. In case there are dropouts, these patients will be replaced and the new patients will be assigned in an identical manner. If a patient drops out of the study at month 6 due to his/her own decision, the patient data will be removed from the trial completely, including any data generated prior to the withdrawal. If a patient passes away during the first 6 months, the data will be used. If the urine probes reveal that a patient from the broccoli sprouts group did not consume the broccoli sprouts daily or if a patient from the placebo group had a high dietary intake of isothiocyanates, the data from these patients will not be used, but the patient will be replaced by a new patient, even if a patient drops out between months 9 to 12. This case will extend the duration of the study. Randomly generated numbers will be allocated to the two groups in balanced permuted blocks using a number generator (validated software, SAS). In order to avoid any possibility of predicting the group allocation, the length of the blocks will be fixed in a separate document and this information will be withheld from the study site. A data sheet that links the group allocation with the randomized number of the patient will be sealed in pre-produced, non transparent envelopes labeled with the arbitrary patient numbers and these envelopes will be used in consecutive order. In order to be able to perform a retrospective control of the compliance to randomization, basic patient data, including the day on which the patient’s number was assigned, will be noted on the data sheet. After completing the trial, all unopened envelopes will be delivered to the data management center for comparison to the allocated, randomized patient numbers and examination and evaluation of the thoroughness of the trial.

The investigators, patients, and assessors of the POUDER trial will be blind to the patients’ study treatment courses. The principal investigator (PI) will be provided with a set of sealed envelopes containing information on patients’ trial drugs, one for each patient number. These envelopes are to be opened only under imperative circumstances (medical indication to know the substance in use by the subject).

### Data management and statistical analysis

The trial investigator or a designated representative (sub-investigator) will document all data acquired during the trial in a case report form (CRF) on the day of the routine visit. All missing or inconsistent data will be reported and inferred. Upon completion, all CRFs must be officially reviewed and signed by either the investigator named in the trial protocol or by a designated sub-investigator. In order to assure the highest data quality and accurate transfer from the CRF to the database, a double data-entry method will be performed. After completing the trial, the PI will retain the original CRFs.

Totality, validity, and plausibility of the data will be examined by validating programs that will generate queries that must be clarified by the investigator. All findings, both clinical and overall survival, imaging of disease stages, quality of life, and biochemical analyses will be noted in the subject’s CRF.

As this is a pilot study, no sample size calculation was performed. A sample size of 20 patients per study group has been deemed sufficient for the study objectives. The sample size of 20 patients per group is speculative at this time and it is based on comparable patient numbers used in similar ongoing and recently completed patient studies with broccoli sprout extracts. For example, the Pittsburgh pilot study evaluating sulforaphane in atypical nevi-precursor lesions enrolled 18 patients (http://clinicaltrials.gov/ct2/show/NCT01568996?term=NCT01568996&rank=1) and another study performed at OHSU Knight Cancer Institute for examination of sulforaphane in treating patients with recurrent prostate cancer (http://clinicaltrials.gov/ct2/show/record/NCT01228084?term=sulforaphane&rank=1) enrolled 20 patients.

Depending on data characteristics, descriptive statistics of the monitored parameters will be presented as mean values and SD or median values and IQR. Descriptive *P*-values of the *t*-test or the Mann-Whitney *U*-test comparing the treatment groups will be given, together with the corresponding 95% CI. Categorical parameters will be presented as relative and absolute frequencies, and comparisons will be performed using the Fisher exact test.

Survival curves will be constructed using the Kaplan-Meier estimator. The descriptive *P*-value of the log-rank test will be used to compare the survival curves of the treatment groups. Estimated median survival times and the one-year survival rates with 95% CI will be given.SAS software (Release 9.1, SAS Institute, Inc. Cary, NC, USA) and Microsoft Office Excel 97® (Redmond, Washington, USA) will be used for the statistical analysis and graphical presentations.

### Adverse and serious adverse events

Any manifestation of symptoms or conditions, laboratory parameter alteration, or recurrence or aggravation of existing co-morbidity that correlate with the use of an investigational or medicinal substance or product constitutes an adverse event (AE). A serious adverse event (SAE) is defined as an AE that is life threatening and necessitates hospitalization and/or prolonged medical treatment until stabilization. The occurrence of all events that can be classified as adverse will be recorded and its clinical course will be followed until resolution and stabilization. Events associated with the primary diagnosis, the surgical procedure itself, or complications related to the palliative chemotherapy will not be registered as SAEs unless deemed so by the investigator. All SAEs will be documented and reported to the PI (author PS) within 24 hours or during the following calendar working day, at the latest. The PI will then report the newly documented SAEs to the ethics committee and the investigators. Apart from meteorism, which is a common phenomenon associated with cabbage, a very low risk for AEs is expected during this trial.

### Assurance of quality

The principles of the International Conference on Harmonisation-Good Clinical Practice (ICH-GCP) guidelines [32], the regulations of the Declaration of Helsinki, [33] and local legal and regulatory requirements will be adhered to at all times during the trial period. Medical confidentiality and accordance with the German Federal Data Protection Act will also be observed [34].

## Discussion

Since ancient times, cabbages and other extracts of the botanic family *Brassicacea* have been used as natural antibiotics and key components of antiviral drugs and antimycotics. Members of the *Brassicacea* family contain glycosides that trigger the release of mustard oils after enzymatical hydrolyzation, in case of physical damage of the plant cells. These mustard oils offer protection against microorganisms and fungi. To date, the bioactive function of more than 120 different mustard oils have been documented [35].

With regard to numerous worldwide studies, an inverse relationship between consumption of cruciferous vegetables (including members of the *Brassicacea* family) and the risk of neoplastic diseases has been observed, namely in colorectal, gastric, lung, breast, prostate, bladder, and endometrial cancers [18,19]. The bioactive phytochemicals, sulforaphane and quercetin have been shown to be potent substances that possess chemopreventive and therapeutic properties beneficial in cancers of the intestine, breast [28,29], prostate [17], and pancreas [20]. Sulforaphane, and its inactive glycoside precursor, glucoraphanin, are present in high concentrations in broccoli and its sprouts [18,19]. Sulforaphane is a potent antioxidant that indirectly eliminates free radicals; by increasing the concentration of glutathione, sulforaphane inhibits the formation and accumulation of carcinogen-induced DNA adducts [18,36], a finding that has been demonstrated in several animal studies [25,26]. Furthermore, sulforaphane is of interest as a new therapeutic anti-cancer substance, based on recent experimental studies in which the compound has been shown to target CSCs in models of pancreatic, breast, and prostate cancer [20,21]. In pancreatic cancer, sulforaphane was shown to exhibit its anti-CSC effect through the downregulation of NF-κB activity, which is usually enhanced in active CSCs [18]. Consequently, sulforaphane might be effective in overcoming the resistance of pancreatic cancer cells to chemotherapy, as underlined by recent experimental studies. These studies show that sulforaphane increases the responsiveness of pancreatic CSCs to sorafenib, gemcitabine, cisplatin, doxorubicin, and 5-fluorouracil [20,22,23].

Another bioactive agent is quercetin, a polyphenol flavonoid commonly found in apples, berries, red grapes, onions, and broccoli, [37] with antioxidant, anticarcinogenic, anti-inflammatory and cardioprotective activities [38,39]. Recent reports describe the possible use of quercetin to inhibit the self-renewal and therapy resistance of pancreatic CSCs by affecting clonogenicity, spheroid formation, and aldehyde dehydrogenase isoform 1 (ALDH1) activity, as well as blocking the signaling pathways involved in apoptosis resistance, proliferation, angiogenesis, NF-κB activity, and epithelial-mesenchymal transition (EMT) [27]. It is most effective in combination with sulforaphane both *in vitro* and *in vivo*, without inducing toxic side effects in mice [27]. These findings emphasize the need for dietary intervention studies to elucidate the therapeutic effects of vegetables rich in sulforaphane and quercetin for more successful treatment of therapy-resistant PDA.

Since 2012 a pilot study has been evaluating the dose-dependent effects of broccoli sprout extract on atypical nevi, as precursor lesions and malignant melanoma (clinicaltials.gov identifier: NCT01568996) in an 18-patient collective. Two other ongoing pilot studies are examining the preoperative administration of broccoli sprout extract in patients with transitional cell bladder cancer undergoing surgery (NLM identifier: NCT01108003) and the additive intake of broccoli sprout extract in patients with recurrent prostate cancer (clinicaltrials.gov identifier: NCT01228084).

From the available studies, it can be noted that there is a positive correlation between the consumption of broccoli and an increased efficacy of chemotherapy [27,40]. However, to date this is not based on high-level evidence trials. The POUDER trial will not interfere with any palliative chemotherapy or targeted therapy as these substances typically do not require any food restrictions. Consequently, patients who participate in PDA trials can simultaneously participate in a broccoli nutritional study. However, compounding effects of the intake of broccoli sprouts might have an influence on the results of any parallel trial due to the potential benefit of their application. The present trial has to be regarded as a test of feasibility. Participation in other treatment schemes - regardless of whether or not standard or study substances are applied - was not defined as a contraindication for inclusion. As patients suffering from tumor diseases are often encouraged to make healthy changes in their lifestyle, other phytotherapies may be expected. Yet, the effects of the chosen high dosage of the broccoli sprout extract will probably overpower the effects of any other nutritional substance.

A possible side effect of high broccoli sprout intake may be meteorism, which is a common phenomenon associated with cabbage consumption. Other effects include the prominence of the rotten-egg odor. During the metabolic breakdown of cabbage in the digestive tract, sulfur in the glucosinolates [41] is converted into the rotten-egg chemical, hydrogen sulfide, by gut bacteria. These potential side effects may be uncomfortable, but are considered to be insignificant in the overall context of a promising therapy for an advanced tumor disease.

With regard to sulforaphane, its modus operandi is believed to be as an indirect antioxidant that helps to protect healthy cells from oxidative damage and reduce the short- and long-term harmful effects of cancer treatment [42]. However, concern has been raised that antioxidant supplements may also protect tumor cells during chemotherapy, thereby compromising treatment efficacy [42,43]. This has resulted in controversy over the guidelines for the use of vitamin supplements during cancer treatment. This issue was examined in an experimental human PDA xenograft model in mice. In this setup, sulforaphane did not protect tumor cells; instead, it increased the effect of gemcitabine, cisplatin, doxorubicin, 5-fluorouracil, sorafenib, and TRAIL [20,22,23,27].

In closing, the POUDER trial is the first clinical pilot trial to test the feasibility of an intake of freeze-dried broccoli sprouts as an additive of palliative chemotherapy in surgically non-resectable, advanced pancreatic cancer. Depending on the results of this pilot trial, a hypothesis for a confirmatory trial in the field will be generated and a consecutive RCT is planned.

## Trial status

The POUDER trial has been designed as a single center pilot randomized controlled trial (RCT). The initiation took place at the Department of Surgery, University of Heidelberg in December 2013 after approval by the local ethical committee.

## Abbreviations

AE: adverse event; ALDH1: aldehyde dehydrogenase isoform 1; CA 19-9: carbohydrate antigen 19-9; CEA: carcinoembryonic antigen; CRF: case report form; CSC: cancer stem cells; CT: computed tomography; HPLC: high performance liquid chromatography; MRI: magnetic resonance imaging; NF-κB: Nuclear factor-kappa beta; PDA: pancreatic ductal adenocarcinoma; PI: principal investigator; RCT: randomized controlled trial; SAE: serious adverse event.

## Competing interests

The authors have no competing interests to declare.

## Authors’ contributions

VJL, PH, IH, and PS designed the study. PS performed a quality review to ensure adherence to current guidelines and laws. UH supported the statistical design. TH supported the design of the study. VJL, IH, and PS wrote the manuscript. All authors have read and approved its final version.

## References

[B1] Hariharan D Saied A Kocher HM Analysis of mortality rates for pancreatic cancer across the world HPB (Oxford) 2008 10 58 62 10.1080/13651820701883148 18695761 PMC2504856

[B2] Witkowski ER Smith JK Tseng JF Outcomes following resection of pancreatic cancer J Surg Oncol 2013 107 97 103 10.1002/jso.23267 22991309

[B3] Schmidt J Abel U Debus J Harig S Hoffmann K Herrmann T Bartsch D Klein J Mansmann U Jäger D Capussotti L Kunz R Büchler MW Open-label, multicenter, randomized phase III trial of adjuvant chemoradiation plus interferon Alfa-2b versus fluorouracil and folinic acid for patients with resected pancreatic adenocarcinoma J Clin Oncol 2012 30 4077 4083 10.1200/JCO.2011.38.2960 23008325

[B4] Siegel R Naishadham D Jemal A Cancer statistics, 2013 CA Cancer J Clin 2013 63 11 30 10.3322/caac.21166 23335087

[B5] Tuveson DA Neoptolemos JP Understanding metastasis in pancreatic cancer: a call for new clinical approaches Cell 2012 148 21 23 10.1016/j.cell.2011.12.021 22265397

[B6] Li D Xie K Wolff R Abbruzzese JL Pancreatic cancer Lancet 2004 363 1049 1057 10.1016/S0140-6736(04)15841-8 15051286

[B7] Oberstein PE Saif MW First-line treatment for advanced pancreatic cancer. Highlights from the “2011 ASCO Gastrointestinal Cancers Symposium”. San Francisco, CA, USA. January 20-22, 2011 JOP 2011 12 96 100 21386629

[B8] Lapidot T Sirard C Vormoor J Murdoch B Hoang T Caceres-Cortes J Minden M Paterson B Caligiuri MA Dick JE A cell initiating human acute myeloid leukaemia after transplantation into SCID mice Nature 1994 367 645 648 10.1038/367645a0 7509044

[B9] Reya T Morrison SJ Clarke MF Weissman IL Stem cells, cancer, and cancer stem cells Nature 2001 414 105 111 10.1038/35102167 11689955

[B10] Rasheed ZA Kowalski J Smith BD Matsui W Concise review: emerging concepts in clinical targeting of cancer stem cells Stem Cells 2011 29 883 887 10.1002/stem.648 21509907 PMC3355871

[B11] Al-Hajj M Wicha MS Benito-Hernandez A Morrison SJ Clarke MF Prospective identification of tumorigenic breast cancer cells Proc Natl Acad Sci USA 2003 100 3983 3988 10.1073/pnas.0530291100 12629218 PMC153034

[B12] Singh SK Hawkins C Clarke ID Squire JA Bayani J Hide T Henkelman RM Cusimano MD Dirks PB Identification of human brain tumour initiating cells Nature 2004 432 396 401 10.1038/nature03128 15549107

[B13] Simeone DM Pancreatic cancer stem cells: implications for the treatment of pancreatic cancer Clin Cancer Res 2008 14 5646 5648 10.1158/1078-0432.CCR-08-0584 18794070

[B14] Abbott A Cancer: the root of the problem Nature 2006 442 742 743 10.1038/442742a 16915262

[B15] Forman D Burley V Cade J Greenwood D Moreton J Chan D Tu Y-K Gordon I Thomas J McColl K The associations between food, nutrition and physical activity and the risk of pancreatic cancer and underlying mechanisms World Cancer Research Fund. Food, nutrition, physical activity, and the prevention of cancer: a global perspective 2007 Washington, DC: AICR

[B16] Kirsh VA Peters U Mayne ST Subar AF Chatterjee N Johnson CC Hayes RB Prostate, lung, colorectal and ovarian cancer screening trial, prospective study of fruit and vegetable intake and risk of prostate cancer J Natl Cancer Inst 2007 99 1200 1209 10.1093/jnci/djm065 17652276

[B17] Richman EL Carroll PR Chan JM Vegetable and fruit intake after diagnosis and risk of prostate cancer progression Int J Cancer 2012 131 201 210 10.1002/ijc.26348 21823116 PMC3310254

[B18] Herr I Lozanovski V Houben P Schemmer P Büchler MW Sulforaphane and related mustard oils in focus of cancer prevention and therapy Wien Med Wochenschr 2013 163 80 88 10.1007/s10354-012-0163-3 23224634

[B19] Fahey JW Zhang Y Talalay P Broccoli sprouts: an exceptionally rich source of inducers of enzymes that protect against chemical carcinogens Proc Natl Acad Sci USA 1997 94 10367 10372 10.1073/pnas.94.19.10367 9294217 PMC23369

[B20] Kallifatidis G Rausch V Baumann B Apel A Beckermann BM Groth A Mattern J Li Z Kolb A Moldenhauer G Altevogt P Wirth T Werner J Schemmer P Büchler MW Salnikov AV Herr I Sulforaphane targets pancreatic tumour-initiating cells by NF-kappaB induced antiapoptotic signalling Gut 2009 58 949 963 10.1136/gut.2008.149039 18829980

[B21] Li Y Wicha MS Schwartz SJ Sun D Implications of cancer stem cell theory for cancer chemoprevention by natural dietary compounds J Nutr Biochem 2011 22 799 806 10.1016/j.jnutbio.2010.11.001 21295962 PMC3248810

[B22] Rausch V Liu L Kallifatidis G Baumann B Mattern J Gladkich J Wirth T Schemmer P Büchler MW Zöller M Salnikov AV Herr I Synergistic activity of sorafenib and sulforaphane abolishes pancreatic cancer stem cell characteristics Cancer Res 2010 70 5004 5013 10.1158/0008-5472.CAN-10-0066 20530687

[B23] Kallifatidis G Labsch S Rausch V Mattern J Gladkich J Moldenhauer G Büchler MW Salnikov AV Herr I Sulforaphane increases drug-mediated cytotoxicity towards cancer stem-like cells of pancreas and prostate Mol Ther 2011 19 188 195 10.1038/mt.2010.216 20940707 PMC3017446

[B24] Silverman DT Swanson CA Gridley G Wacholder S Greenberg RS Brown LM Hayes RB Swanson GM Schoenberg JB Pottern LM Schwartz AG Fraumeni JF Jr Hoover RN Dietary and nutritional factors and pancreatic cancer: a case-control study based on direct interviews J Natl Cancer Inst 1998 90 1710 1719 10.1093/jnci/90.22.1710 9827525

[B25] Bertl E Bartsch H Gerhauser C Inhibition of angiogenesis and endothelial cell functions are novel sulforaphanemediated mechanisms in chemoprevention Mol Cancer Ther 2006 5 575 585 10.1158/1535-7163.MCT-05-0324 16546971

[B26] Juge N Mithen RF Traka M Molecular basis for chemoprevention by sulforaphane: a comprehensive review Cell Mol Life Sci 2007 64 1105 1127 10.1007/s00018-007-6484-5 17396224 PMC11136334

[B27] Zhou W Kallifatidis G Baumann B Rausch V Mattern J Gladkich J Giese N Moldenhauer G Wirth T Büchler MW Salnikov AV Herr I Dietary polyphenol quercetin targets pancreatic cancer stem cells Int J Oncol 2010 37 551 561 20664924 10.3892/ijo_00000704

[B28] Cornblatt BS Ye L Dinkova-Kostova AT Erb M Fahey JW Singh NK Chen MS Stierer T Garrett-Mayer E Argani P Davidson NE Talalay P Kensler TW Visvanathan K Preclinical and clinical evaluation of sulforaphane for chemoprevention in the breast Carcinogenesis 2007 28 1485 1490 10.1093/carcin/bgm049 17347138

[B29] Seow A Yuan JM Sun CL Van Den Berg D Lee HP Yu MC Dietary isothiocyanates, glutathione S-transferase polymorphisms and colorectal cancer risk in the Singapore Chinese Health Study Carcinogenesis 2002 23 2055 2061 10.1093/carcin/23.12.2055 12507929

[B30] Shapiro TA Fahey JW Dinkova-Kostova AT Holtzclaw WD Stephenson KK Wade KL Ye L Talalay P Safety, tolerance, and metabolism of broccoli sprout glucosinolates and isothiocyanates: a clinical phase I study Nutr Cancer 2006 55 53 62 10.1207/s15327914nc5501_7 16965241

[B31] Egner PA Chen JG Wang JB Wu Y Sun Y Lu JH Zhu J Zhang YH Chen YS Friesen MD Jacobson LP Muñoz A Ng D Qian GS Zhu YR Chen TY Botting NP Zhang Q Fahey JW Talalay P Groopman JD Kensler TW Bioavailability of Sulforaphane from two broccoli sprout beverages: results of a short-term, cross-over clinical trial in Qidong, China Cancer Prev Res (Phila) 2011 4 384 395 10.1158/1940-6207.CAPR-10-0296 21372038 PMC3076202

[B32] International Conference on Harmonisation of Technical Requirements for Registration of Pharmaceuticals for Human Use (ICH) Guideline E6: Note for Guidance on good clinical practice (GCP) http://www.nus.edu.sg/irb/Articles/ICH%20GCP%20E6.pdf

[B33] World Medical Association Declaration of Helsinki Recommendations Guiding Physicians in Biomedical Research Involving Human Subjects, Adopted by the 18th World Medical Assembly Helsinki, Finland, June 1964, amended by 64th WMA General Assembly, Fortaleza Brazil 2013 http://www.wma.net/en/30publications/10policies/b3/

[B34] Federal Data Protection Act (BDSG) In the version promulgated on 14 January 2003 (Federal Law Gazette I, p. 66), last amended by Article 1 of the Act of 14 August 2009 (Federal Law Gazette I, p. 2814) http://www.bfdi.bund.de/EN/DataProtectionActs/Artikel/BDSG_idFv01092009.pdf?__blob=publicationFile

[B35] Fahey JW Zalcmann AT Talalay P The chemical diversity and distribution of glucosinolates and isothiocyanates among plants Phytochemistry 2001 56 5 51 10.1016/S0031-9422(00)00316-2 11198818

[B36] Shishu Singla AK Kaur IP Inhibition of mutagenicity of food-derived heterocyclic amines by sulphoraphenean isothiocyanate isolated from radish Planta Med 2003 69 184 186 10.1055/s-2003-37713 12624832

[B37] Watzl B Leitzmann C Bioaktive Substanzen in Lebensmitteln 2005 3 Stuttgart: Hippokrates Verlag

[B38] Williamson G Manach C Bioavailability and bioefficacy of polyphenols in humans, II. Review of 93 intervention studies Am J Clin Nutr 2005 81 243 255 10.1093/ajcn/81.1.243S 15640487

[B39] Ramos S Effects of dietary flavonoids on apoptotic pathways related to cancer chemoprevention J Nutr Biochem 2007 18 427 442 10.1016/j.jnutbio.2006.11.004 17321735

[B40] Adikrisna R Tanaka S Muramatsu S Aihara A Ban D Ochiai T Irie T Kudo A Nakamura N Yamaoka S Arii S Identification of pancreatic cancer stem cells and selective toxicity of chemotherapeutic agents Gastroenterology 2012 143 234 245 10.1053/j.gastro.2012.03.054 22510202

[B41] Halkier BA Gershenzon J Biology and biochemistry of glucosinolates Annu Rev Plant Biol 2006 57 303 333 10.1146/annurev.arplant.57.032905.105228 16669764

[B42] Lawenda BD Kelly KM Ladas EJ Sagar SM Vickers A Blumberg JB Should supplemental antioxidant administration be avoided during chemotherapy and radiation therapy? J Natl Cancer Inst 2008 100 773 783 10.1093/jnci/djn148 18505970

[B43] D’Andrea GM Use of antioxidants during chemotherapy and radiotherapy should be avoided CA Cancer J Clin 2005 55 319 321 10.3322/canjclin.55.5.319 16166076

